# Sleep satisfaction and problematic smartphone use among adolescents in the Republic of Korea: The mediating roles of anxiety and loneliness

**DOI:** 10.1371/journal.pone.0354423

**Published:** 2026-07-24

**Authors:** Jiyeon Kim, Myongsun Cho

**Affiliations:** 1 Department of Nursing, Semyung University, Jecheon-si, Republic of Korea; 2 Department of Nursing, Kangwon National University, Wonju-si, Republic of Korea; Minnan Normal University, CHINA

## Abstract

Problematic smartphone use (PSU) has become a growing public health concern among adolescents, with global prevalence estimates ranging from 10% to 30%. Sleep dissatisfaction has been identified as a potential risk factor for PSU. However, the psychological mechanisms underlying this association remain unclear. This study examined the relationship between sleep satisfaction and PSU among Korean adolescents, and investigated the mediating roles of anxiety and loneliness. Data were collected from 48,829 adolescents who were categorized into three groups: general users (72.1%), potential-risk users (24.8%), and high-risk users (3.1%). Group differences were examined using chi-square tests and one-way analysis of variance (ANOVA). Multivariate logistic regression analyses were conducted to identify the factors associated with PSU risk. Mediation analyses were conducted using a counterfactual framework to estimate the total, natural direct, and natural indirect effects of sleep satisfaction on PSU risk through anxiety and loneliness. Increased PSU risk was significantly associated with being female, high smartphone usage time (≥4 hours/day), and reporting poor perceived health. High-risk users reported markedly longer daily smartphone use (7.01 ± 0.12 hours) and significantly higher anxiety and loneliness scores compared to other groups (*p* < .001). Poor sleep satisfaction and insufficient physical activity were also significantly associated with higher PSU risk. Mediation analyses indicated significant natural indirect effects through anxiety and loneliness, with proportions mediated of 30.0% and 15.2%, respectively. Sleep dissatisfaction is significantly associated with problematic smartphone use among adolescents, and this relationship is partly explained by heightened anxiety and loneliness. Interventions aimed at improving sleep satisfaction and addressing emotional vulnerability may be effective strategies for reducing the PSU risk in this population.

## Introduction

Problematic smartphone use (PSU) refers to a condition in which individuals cannot self-regulate their smartphone use, leading to an excessive increase in the urge to use it and causing negative effects on their daily lives, academics, and social relationships [1,2]. PSU should be distinguished from merely frequent or prolonged smartphone use, because problematic use reflects functional impairment and compulsive tendencies rather than duration alone [3]. This distinction is also supported by evidence showing that PSU symptoms are more strongly associated with behavioral difficulties, poorer quality of life, and school-related problems than smartphone usage time [4]. Adolescence may be a particularly vulnerable developmental period for PSU because self-regulatory capacity, reward sensitivity, and emotional regulation systems are still maturing. This makes adolescents more susceptible to dysregulated digital behaviors when they encounter emotional or interpersonal stressors [5].

The prevalence of PSU also varies significantly depending on the country and target group. Research has pointed out that over 85% of Korean adolescents use smartphones for more than two hours a day, with a significant number using them for more than four hours a day [6]. Furthermore, a study on adolescents and young adults in Austria reported that approximately 38.1% belonged to the PSU risk group [7], and another study on Malaysian adolescents reported a PSU prevalence rate of 43.5% [8].

These findings indicate that problematic digital engagement is not confined to one cultural setting but represents a broader developmental and public health concern. PSU in adolescence, is associated with psychosocial maladjustment and may co-occur with internalizing symptoms, hence, more precise identification of its modifiable antecedents is needed to inform prevention and intervention strategies [9]. Therefore, a stronger theoretical understanding of PSU is necessary.

PSU in the present study, is conceptualized not simply as a habit of excessive use, but as a maladaptive behavior that may emerge when adolescents use smartphones to compensate for emotional discomfort, unmet interpersonal needs, or weakened self-regulatory functioning. This perspective is consistent with the Compensatory Internet Use Theory, which proposes that problematic online behavior can arise when individuals attempt to cope with negative emotions or stressful life circumstances through digital engagement [5]. It is also consistent with the Interaction of Person-Affect-Cognition-Execution (I-PACE) model, which explains problematic technology use as the result of interactions among personal predispositions, affective responses, cognitive biases, and impaired executive control [5]. Anxiety in related conceptual work, has been identified as a particularly relevant affective state that may intensify reassurance seeking, avoidance, fear of missing out, and repetitive checking behaviors through smartphones, thereby increasing PSU risk [10]. These frameworks suggest that adolescents may not develop PSU simply because they have access to smartphones, but because smartphones may become a readily available tool for coping with distress under conditions of weakened emotional and behavioral regulation. Sleep should not be treated only as a consequence of PSU but also as a plausible upstream vulnerability factor, within this broader theoretical framework. Previous studies have consistently shown that PSU is associated with poorer sleep quality and sleep-related problems [11,12], and reciprocal influences between sleep and PSU are likely. However, much of the existing literature has emphasized the pathway from PSU to sleep disturbance, particularly through nighttime arousal, delayed sleep onset, and difficulty disengaging from devices [13]. Although this line of research is important, it does not fully explain why some adolescents become psychologically dependent on smartphones in the first place. Unsatisfactory sleep may deplete emotional resources, impair self-regulation, and heighten interpersonal sensitivity, from a psychosocial vulnerability perspective. This increases the likelihood that adolescents turn to smartphones in maladaptive ways.

Therefore, this study focuses on sleep satisfaction—a subjective indicator that may capture more than objective sleep duration alone. Subjective sleep evaluations reflect how restorative one’s sleep feels and whether sleep is perceived as sufficient for daytime functioning, emotional recovery, and coping. Prior adolescent sleep research has noted that subjective sleep complaints deserve direct attention, because they may not fully overlap with objective sleep parameters, and often reflect broader psychological burden [14]. Accordingly, low sleep satisfaction may indicate not only poor sleep experience itself but also diminished recovery capacity, fatigue, irritability, and weakened emotion regulation resources. Thus, sleep satisfaction is theoretically meaningful as a potential antecedent condition that may increase vulnerability to maladaptive coping behaviors such as PSU.

This distinction is especially important because PSU should also be differentiated from post-bedtime smartphone use. Smartphone use in bed or before sleep is a behavior defined by timing and context, whereas PSU is a broader clinical-behavioral construct involving persistent loss of control and functional impairment. Research on restricting while-in-bed smartphone use shows that such bedtime behavior is closely related to sleep quality through mechanisms such as pre-sleep cognitive arousal [13]. However, bedtime use does not necessarily imply PSU. An adolescent may use a smartphone at night out of habit, convenience, or situational preference, whereas PSU implies a more generalized pattern of dysregulated, compulsive, and impairment-related use. Therefore, to understand PSU in adolescents, it is necessary to move beyond behavior-specific explanations and examine whether subjective sleep dissatisfaction is indirectly associated with PSU through emotional and social vulnerability.

Based on this reasoning, the present study proposes loneliness and anxiety as two parallel mediators linking sleep satisfaction to PSU. First, loneliness represents an important social-affective pathway. Adolescents who feel socially disconnected may use smartphones to seek reassurance, belonging, distraction, or online social compensation. Prior studies have shown that loneliness is closely related to problematic online behavior, and coping-oriented digital use has been suggested as one mechanism through which interpersonal difficulties become linked to problematic internet use [15]. Loneliness in adolescent PSU research, has also been identified as a meaningful intervention target, with evidence showing that reductions in loneliness accompany improvements in PSU symptoms [16]. Thus, when low sleep satisfaction is associated with fatigue, irritability, and social withdrawal, it may also be associated with heightened perceived loneliness, which in turn may increase the likelihood of maladaptive smartphone engagement.

Second, anxiety represents an emotional pathway that is also theoretically central to PSU. Conceptual and empirical work has shown that anxiety symptoms are consistently associated with PSU severity, with possible mechanisms including reassurance seeking, fear of missing out, avoidance of offline discomfort, and repetitive checking behaviors [10]. Psychological distress in adolescent samples, has been linked to PSU through maladaptive cognitive-affective processes, providing empirical support for both the compensatory and I-PACE perspectives [5]. Network-based evidence further suggests that PSU and anxiety symptoms occupy closely connected positions within broader adolescent psychopathology structures, underscoring the clinical importance of this association [9]. Adolescents with low sleep satisfaction may experience heightened worry, tension, and reduced emotional control during the day, making smartphone use more reinforcing as a short-term regulation strategy and thereby increasing PSU risk.

Importantly, loneliness and anxiety are not competing explanations but complementary mediating mechanisms. Sleep dissatisfaction may simultaneously undermine adolescents’ sense of emotional stability and their sense of social connectedness. In turn, both internal distress and interpersonal disconnection may motivate compensatory smartphone use. This mediation logic is also consistent with broader longitudinal evidence showing that psychosocial vulnerability factors can precede later PSU through intermediate regulatory or relational mechanisms, rather than PSU arising solely from exposure or habit [17]. Therefore, examining loneliness and anxiety as parallel mediators allows a more theoretically integrated explanation of how subjective sleep dissatisfaction may become linked to PSU in adolescence.

The present study is guided by a psychosocial vulnerability framework in which low sleep satisfaction is conceptualized as an upstream condition that may be associated with emotional vulnerability, represented by anxiety, and social vulnerability, represented by loneliness, thereby increasing the likelihood of PSU. This framework integrates adolescent sleep research with compensatory and affect-regulation perspectives of problematic technology use. It also provides a clearer theoretical rationale for why sleep satisfaction should be considered not merely an outcome of smartphone behavior but also a plausible antecedent condition in adolescent PSU.

Accordingly, the theoretical framework of this study can be summarized as follows:

Low sleep satisfaction → higher loneliness → higher PSU

Low sleep satisfaction → higher anxiety symptoms → higher PSU

Although reverse or reciprocal pathways remain plausible, especially because PSU itself may also worsen sleep, the study adopts this direction as a theory-driven mediation model appropriate for testing whether subjective sleep dissatisfaction is indirectly associated with PSU through adolescents’ emotional and social vulnerability. By doing so, this study seeks to clarify a higher-level public health mechanism of PSU and to provide evidence that both sleep-related and psychosocial factors may serve as intervention targets in adolescent PSU prevention.

### Research hypotheses

H1. Lower sleep satisfaction will be associated with higher odds of PSU risk.
H2. Lower sleep satisfaction will be associated with higher loneliness.
H3. Lower sleep satisfaction will be associated with higher anxiety symptoms.
H4. Loneliness will significantly mediate the association between sleep satisfaction and PSU risk.
H5. Anxiety symptoms will significantly mediate the association between sleep satisfaction and PSU risk.

### Null hypotheses

H0-1. Sleep satisfaction will not be associated with PSU risk.
H0-2. Sleep satisfaction will not be associated with loneliness.
H0-3. Sleep satisfaction will not be associated with anxiety symptoms.
H0-4. The indirect effect of sleep satisfaction on PSU risk through loneliness will be zero.
H0-5. The indirect effect of sleep satisfaction on PSU risk through anxiety symptoms will be zero.

Thus, this study foregrounds the role of sleep satisfaction in explaining adolescent PSU and comprehensively examines the psychosocial pathways of loneliness and anxiety.

## Materials and methods

### Participants

This study is a secondary analysis of data from the 2023 Korean Youth Risk Behavior Survey (KYRBS), a nationally representative school-based survey of Korean adolescents. This study was reported in accordance with the Strengthening the Reporting of Observational Studies in Epidemiology (STROBE in S1 Checklist) checklist. The KYRBS is a government-approved national statistical survey (approval number: 117058) jointly administered by the Korea Disease Control and Prevention Agency (KDCA) and Ministry of Education pursuant to Article 19 of the National Health Promotion Act [18]. It is an anonymous, self-administered, web-based survey that has been conducted annually since 2005. The survey uses a nationally representative sample of middle and high school students, ranging from the first year of middle school to the third year of high school. It collects information on a wide range of health-related behaviors, including smoking, alcohol use, physical activity, dietary habits, and mental health, to support national health monitoring and policy development [18]. The data used in this study were de-identified and publicly available for research purposes on the official KDCA website (https://kdca.go.kr/yhs/yhs/main.do). The authors accessed the data for research purposes on 31/03/2026.

The KYRBS target population included middle and high school students nationwide. The survey employed a complex sampling design that incorporated stratification, clustering, and multistage sampling, to ensure national representativeness. A total of 800 schools (400 middle schools and 400 high schools, including public and private institutions) were selected from 17 metropolitan cities and provinces. The sampling frame included both public and private schools. Within each selected school, one or more classes were randomly selected, and all students in the selected classes were invited to participate.

The KYRBS is an anonymous school-based survey conducted by a national authority, hence, the present researchers used only de-identified, publicly available secondary data and had no access to personally identifiable information. Prior to participation, students were provided with information regarding the survey purpose, procedures, voluntary nature of participation, anonymity, and confidentiality. Students participated only if they agreed to complete the survey. The survey was completed anonymously through a self-administered web-based questionnaire using school-provided computers or devices. According to the survey protocol, participation was voluntary, and students could refuse or discontinue participation without penalty. No personally identifiable information was collected in the dataset used for this study. Based on the publicly available survey documentation, no individual-level incentives or deterrents related to participation were reported.

**Fig 1:**
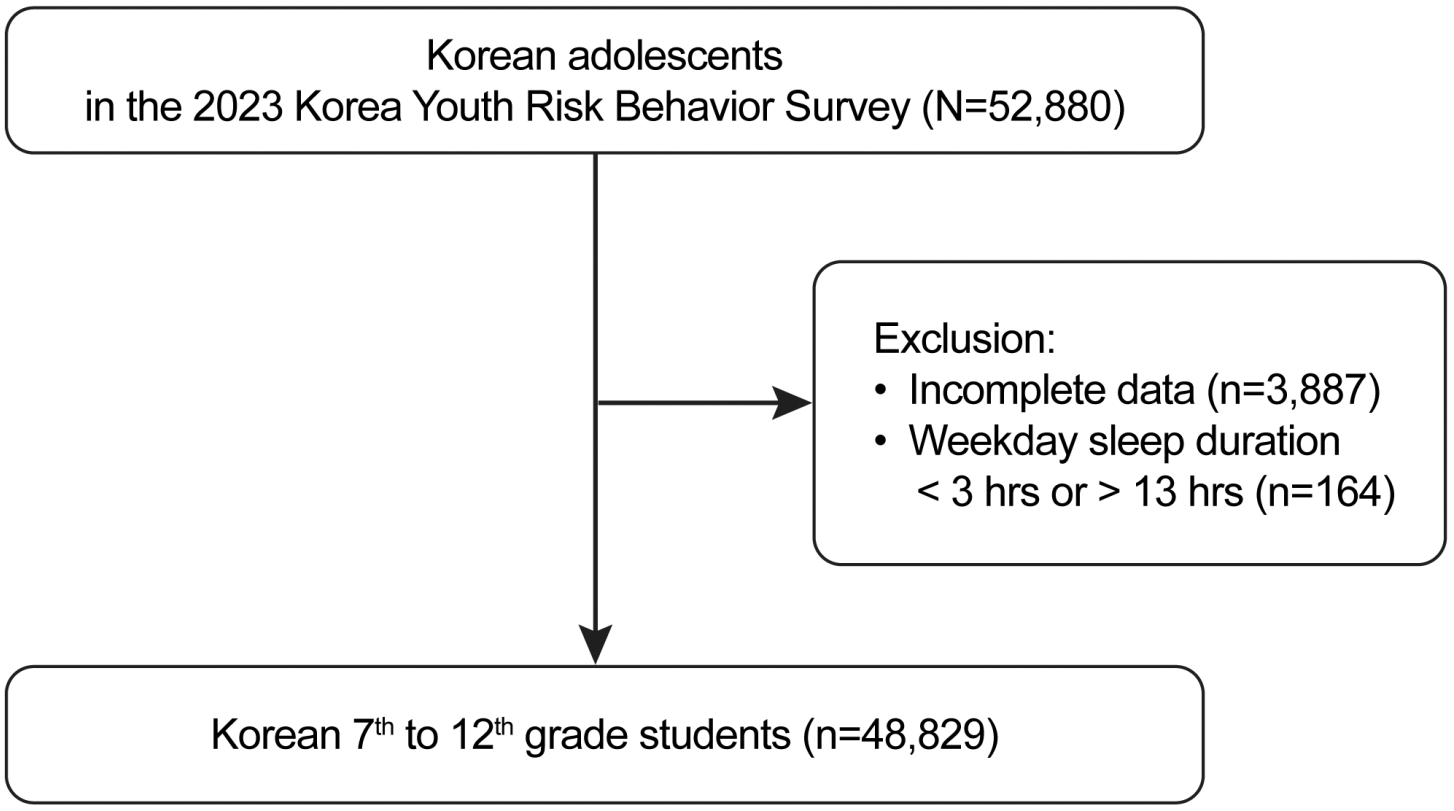
Flowchart of Study Participant Selection.

The average survey completion time was approximately 45–50 minutes. A total of 52,880 students participated in the 2023 KYRBS, with a response rate of 92.5%. 3,887 participants with missing sleep duration data and 164 participants with implausible sleep duration values, defined as less than 3 hours or more than 13 hours per weekday, were excluded from the present study. The final analytic sample comprised 48,829 adolescents (Fig 1).

The KYRBS relies on self-reported responses, hence, the possibility of recall bias, reporting bias, and social desirability bias cannot be completely excluded. The available sociodemographic and health-related variables were included as covariates in the adjusted analyses, to reduce potential confounding. These included sex, school grade, academic achievement, perceived economic status, living arrangement, perceived health status, and other relevant variables available in the dataset. However, some contextual and individual factors, such as detailed family structure, parental monitoring, school-level characteristics, sexual identity, cultural background, deprivation or inequality indicators, and clinically diagnosed comorbid mental health conditions, were not available in the secondary dataset and therefore could not be fully adjusted for in this study.

## Measures

### Problematic smartphone use (PSU)

PSU was assessed using the Problematic Smartphone Use Screening Tool (PSS), a validated instrument used in the Korea Youth Risk Behavior Survey. The PSS was developed based on the Korean Smartphone Overdependence Scale (S-Scale) and was designed to capture key components of addictive behavior, including impaired control, salience, and negative consequences [19]. This conceptual framework is consistent with previous research defining PSU in terms of impaired self-regulation and functional impairment rather than simple usage time [4].

The scale consists of 10 items, including three for assessing impaired control, three for assessing salience, and four for assessing problematic consequences. Each item is rated on a 4-point Likert scale ranging from 1 (not at all) to 4 (very much), yielding a total score ranging from 10 to 40, with higher scores indicating greater PSU severity.

Based on established criteria, participants were classified into three groups: general users (< 23), potential-risk users (23–30), and high-risk users (≥ 31) [18]. PSU risk was operationalized as a binary indicator by combining the potential-risk and high-risk groups. This decision was made for three reasons. First, the PSS was originally designed as a screening tool to identify adolescents at elevated risk of smartphone overdependence in population-based surveillance, making threshold-based classification appropriate for public health research. Second, prior psychometric and screening studies have shown that cutoff-based approaches can validly distinguish adolescents with clinically meaningful PSU risk from lower-risk users [20]. Third, Korean adolescent studies using national survey data have similarly adopted a comparison between general users and combined-risk users, supporting the practical relevance of this binary operationalization for identifying vulnerable groups in need of prevention or intervention [21]. In addition, previous work has suggested that threshold-based classification may be useful when the goal is to identify levels of problematic use associated with adverse psychosocial correlates, although not all PSU instruments are equally suited for diagnostic purposes [22]. Dichotomizing PSU should be interpreted as a risk-screening approach rather than a full representation of the dimensional nature of PSU. Although similar association patterns were observed in preliminary analyses using continuous PSS scores, we maintained a dichotomous outcome variable because the primary objective of this study was to identify adolescents at potential risk of smartphone-related problems. The internal consistency of the scale used in this study was high (Cronbach’s α = 0.907).

### Sleep satisfaction and sleep-related characteristics

The primary exposure was sleep satisfaction, assessed using a single self-reported item: “In the past 7 days, do you think your sleep was sufficient to recover from fatigue?” Responses were recorded on a 5-point Likert scale (1 = not at all sufficient to 5 = very sufficient), with higher scores indicating greater perceived restorative sleep. This item was treated as a global subjective indicator of recent restorative sleep, and not as a measure of multidimensional sleep health. Contemporary sleep-health models, including the RU-SATED, define sleep as a multidimensional construct encompassing regularity, satisfaction, alertness, timing, efficiency, and duration; therefore, a single item cannot capture these domains comprehensively [23,24]. Rather, we used this measure to reflect the adolescents’ overall appraisal of whether their recent sleep was sufficient and restorative. Prior research suggests that single-item sleep satisfaction or overall sleep quality measures can function as pragmatic summary indicators and are meaningfully related to sleep habits and mental health correlates [25,26].

Weekday sleep duration (SDW) was derived from self-reported weekday bedtime and wake time and entered as a continuous covariate. Weekend catch-up sleep was calculated as weekend sleep duration minus SDW and categorized as no catch-up sleep (≤0 h) versus catch-up sleep (>0 h). These variables were included because sleep quantity and compensatory weekend sleep behavior are related to, but conceptually distinct from, subjective sleep satisfaction [27,28]. Adjusting for SDW and catch-up sleep allowed us to estimate the association of sleep satisfaction with the outcome conditional on habitual weekday sleep quantity and compensation patterns, rather than as a total effect of all sleep-related characteristics combined.

As the sleep satisfaction item was explicitly anchored to the past seven days, associations with measures using different reference periods, such as the Perceived Stress Scale, were interpreted cautiously. The observed associations should be understood as cross-sectional associations between recent sleep satisfaction and the comparison measure, and not as evidence of tightly synchronized week-specific processes, in the absence of identical time-locking across measures. This temporal mismatch was considered a measurement limitation [29].

### Psychosocial factors (anxiety and loneliness)

Anxiety was assessed using the seven-item Generalized Anxiety Disorder Scale (GAD-7), a widely used and validated instrument for measuring generalized anxiety symptoms. Each item is rated on a 4-point Likert scale ranging from 0 (not at all) to 3 (nearly every day), resulting in a total score ranging from 0 to 21, with higher scores indicating greater anxiety severity [30]. The internal consistency of the scale used in this study was high (Cronbach’s α = 0.907).

Loneliness was assessed using a single item: “How often did you feel lonely during the past 12 months?” Responses were rated on a 5-point Likert scale ranging from 1 (never) to 5 (always), with higher scores indicating greater loneliness. This item was used as a brief global measure of loneliness. Previous studies have shown that single-item loneliness measures have acceptable validity and reliability in large samples [31]. Since this item referred to the past 12 months, its association with variables assessed over shorter periods should be interpreted cautiously.

### Covariates

Covariates were selected beforehand using a theory-informed causal framework, rather than by the routine inclusion of all available variables, to reduce the risks of overadjustment, unstable estimates, and collider bias [32–34]. The primary adjustment set included variables considered plausible pre-exposure confounders of the association between sleep satisfaction and problematic smartphone use: sex (male/female), school level (middle school/high school), subjective economic status (high/middle/low), residential area (county/medium-sized city/metropolitan area), and subjective health status (poor/fair/good). Current smoking, alcohol use, and physical activity were also included, as these health-related behaviors may be associated with sleep-related experiences or problematic smartphone use.

Current smoking was defined as smoking at least one cigarette on one or more days during the past 30 days, and was categorized as non-smoker or current smoker. Current alcohol use was defined as consuming at least one alcoholic drink on one or more days during the past 30 days and was categorized as non-drinker or current drinker. Physical activity was assessed as the number of days with at least 60 minutes of moderate- or vigorous-intensity physical activity during the past seven days and was categorized as < 5 days or ≥ 5 days.

Average daily smartphone use and sleep duration variables were not included in the primary models. These variables may represent downstream behavioral correlates, proxies, or potential mediators of the studied associations, and indiscriminate adjustment for such variables may introduce bias or obscure the association of interest [34–36]. The average daily smartphone use was calculated as the weighted mean of self-reported weekday and weekend smartphone use:

average daily smartphone use = [(weekday usage time × 5) + (weekend usage time × 2)] / 7. It was categorized as <4 hours/day or ≥4 hours/day, for descriptive purposes.

Weekday sleep duration was derived from self-reported weekday bedtime and wake-up time. Weekend catch-up sleep was calculated as weekend sleep duration minus weekday sleep duration and categorized as no catch-up sleep (≤0 h) or catch-up sleep (>0 h). Since sleep duration and smartphone use may lie on pathways linking sleep satisfaction with psychosocial outcomes, these variables were examined only in the sensitivity analyses (S1 Table) and not in the primary confounder-adjusted models [33,34]. This approach is consistent with the recommendations for tailoring adjustment sets to the specific exposure-outcome contrast under study rather than mutually adjusting for all available risk factors [32,36].

### Statistical analysis

All statistical analyses were performed using SAS version 9.4 (SAS Institute Inc., Cary, NC, USA). Since the Korea Youth Risk Behavior Survey employs a complex sampling design, all analyses were performed using appropriate sampling weights, strata, and cluster variables to account for the complex survey structure and ensure nationally representative estimates.

Descriptive statistics were used to summarize participant characteristics according to the PSU risk groups. Weighted percentages and means with standard errors were calculated, and group differences were assessed using the Rao–Scott chi-square test for categorical variables and weighted analysis of variance (ANOVA) for continuous variables, with post hoc comparisons performed using Tukey’s method.

Multivariable logistic regression analysis accounting for the complex survey design (including sampling weights, clustering, and stratification) was conducted, and odds ratios (ORs) and 95% confidence intervals (CIs) were reported, to identify the factors associated with PSU risk. PSU was dichotomized into general users and at-risk users (potential- and high-risk groups combined).

A mediation analysis was conducted using the CAUSALMED procedure under a counterfactual framework to estimate the total effect, natural direct effect (NDE), and natural indirect effect (NIE) of sleep satisfaction on problematic smartphone use [37,38]. Anxiety and loneliness were examined in separate mediation models because they represent related but distinct psychosocial constructs. The alternate psychosocial variable was included as a covariate in the corresponding model, to estimate the independent mediating effect of each variable [39,40].

Multicollinearity was assessed using the variance inflation factor (VIF), and no problematic collinearity was identified (all VIFs < 2). Variables potentially located on the causal pathway, including average daily smartphone use and sleep duration, were excluded from the primary adjustment set and examined only in the sensitivity analyses, consistent with recommendations to avoid bias from overadjustment in mediation models [37,41].

## Results

### Characteristics of participants

48,829 adolescents were included in the analysis, in total. Among them, 35,342 (72.1%) were classified as general users, 12,011 (24.8%) as potential-risk users, and 1,476 (3.1%) as high-risk users for PSU.

Significant differences in PSU were observed according to sex, perceived household economic status, residential area, smartphone usage time, smoking, alcohol consumption, physical activity, anxiety, loneliness, perceived health, and sleep-related variables (all p < .001) (Table 1).

**Table 1:** Sociodemographic and Health-Related Characteristics According to Levels of Problematic Smartphone Use (PSU).

Characteristics	n (weighted %)				*p* value	Tukey
	Total	General user	Potential-risk user	High-risk user		
**Subjects**	48829	35342 (72.1)	12011 (24.8)	1476 (3.1)		
**Sex**						
**Male**	24559 (51.2)	18726 (54.1)	5285 (44.6)	548 (38.8)	<.001	
**Female**	24270 (48.8)	16616 (45.9)	6726 (55.4)	928 (61.2)		
**Grade**						
**Middle school**	26207 (50.9)	19003 (51.0)	6420 (50.5)	784 (50.9)	<.774	
**High school**	22622 (49.1)	16339 (49.0)	5591 (49.5)	692 (49.1)		
**Perceived household economic status**						
**High**	20711 (43.5)	15408 (44.7)	4724 (40.7)	579 (39.8)	<.001	
**Middle**	22224 (45.0)	16006 (44.7)	5613 (46.1)	605 (40.6)		
**Low**	5890 (11.5)	3925 (10.6)	1673 (13.2)	292 (19.6)		
**Residential area**						
**Rural area**	3875 (5.9)	2876 (6.1)	903 (5.5)	96 (5.0)	.076	
**Middle-sized cities**	23908 (52.8)	17280 (52.7)	5936 (53.2)	692 (50.9)		
**Metropolis**	21046 (41.3)	15186 (41.2)	5172 (41.3)	688 (44.1)		
**Smartphone usage time**						
**<4h/day**	19498 (40.6)	15659 (45.1)	3582 (30.4)	257 (18.2)	<.001	
**≥4h/day**	29331 (59.4)	19683 (54.9)	8429 (69.6)	1219 (81.8)		
**M ± SD**	5.02 ± 0.03	4.73 ± 0.03^a^	5.62 ± 0.01^b^	7.01 ± 0.12^c^	<.001	a < b < c
**Smoking (last 30 days)**						
**No**	46967 (96.2)	34130 (96.5)	11475 (95.6)	1362 (92.3)	<.001	
**Yes**	1862 (3.8)	1212 (3.5)	536 (4.4)	114 (7.7)		
**Alcohol consumption (last 30 days)**						
**No**	43714 (89.4)	31885 (90.1)	10589 (88.1)	1240 (83.9)	<.001	
**Yes**	5115 (10.6)	3457 (9.9)	1422 (11.9)	236 (16.1)		
**Physical activity**						
**<5days/week**	40256 (82.9)	28564 (81.3)	10402 (87.1)	1290 (87.2)	<.001	
**≥5days/week**	8573 (17.1)	6778 (18.7)	1609 (12.9)	186 (12.8)		
**Anxiety**	11.17 ± 0.03	10.53 ± 0.03^a^	12.63 ± 0.05^b^	15.78 ± 0.18^c^	<.001	a < b < c
**Loneliness (1–5), M ± SD**	2.59 ± 0.01	2.46 ± 0.01^a^	2.87 ± 0.01^b^	3.21 ± 0.03^c^	<.001	a < b < c
**Perceived health (1–5), M ± SD**	3.74 ± 0.01	3.82 ± 0.01^a^	3.56 ± 0.01^b^	3.39 ± 0.03^c^	<.001	a > b > c

### Sleep characteristics and problematic smartphone use

Sleep characteristics differed significantly across the PSU levels (Table 2). Mean sleep satisfaction scores decreased progressively from general users (2.90 ± 0.01) to potential-risk users (2.65 ± 0.01) and high-risk users (2.41 ± 0.03) (*p* < .001).

**Table 2:** Sleep Characteristics According to Levels of Problematic Smartphone Use (PSU).

Characteristics	Total (N = 48829)	General user (n = 35342)	Potential-risk user (n = 12011)	High-risk user (n = 1476)	*p* value	Tukey
Sleep satisfaction (1–5), M ± SD	2.82 ± 0.01	2.90 ± 0.01 ^a^	2.65 ± 0.01 ^b^	2.41 ± 0.03 ^c^	<.001	a > b > c
Weekend catch-up sleep (h),						
*n (weighted %)*						
No catch-up (≤ 0 h)	7224 (14.5)	5421 (15.0)	1601 (13.0)	202 (13.8)	<.001	
Catch-up (> 0 h)	24385 (50.0)	17214 (48.7)	6325 (53.0)	846 (56.8)		
M ± SD	2.35 ± 0.02	2.31 ± 0.02^a^	2.42 ± 0.03^b^	2.53 ± 0.09^c^	<.001	a < b, c
Weekday sleep duration (h), M ± SD	6.25 ± 0.01	6.33 ± 0.01 ^a^	6.08 ± 0.02 ^b^	5.80 ± 0.04 ^c^	<.001	a < b < c

The distribution of weekend catch-up sleep differed significantly according to the PSU level (*p* < .001). High-risk users were more likely to report weekend catch-up sleep (> 0 h) (56.8%) than potential-risk (53.0%) and general users (48.7%). The mean duration of weekend catch-up sleep increased across PSU severity groups (2.31 ± 0.02 hours in general users; 2.42 ± 0.03 hours in potential-risk users; 2.53 ± 0.09 hours in high-risk users).

Weekday sleep duration also showed significant differences (*p* < .001). High-risk users reported the shortest weekday sleep duration (5.80 ± 0.04 hours), followed by potential-risk (6.08 ± 0.02 hours) and general users (6.33 ± 0.01 hours).

### Factors associated with problematic smartphone use

Table 3 presents the results of the multivariable logistic regression analysis of the factors associated with PSU. Higher sleep satisfaction was associated with significantly lower odds of PSU (OR = 0.947, 95% CI: 0.924–0.972, *p* < .001). Longer weekday sleep duration was also inversely associated with PSU (OR = 0.954, 95% CI: 0.934–0.973, *p* < .001). Adolescents reporting weekend catch-up sleep (> 0 hours) had higher odds of PSU compared with those reporting no catch-up sleep (OR = 1.080, 95% CI: 1.012–1.153, *p* = .021).

**Table 3:** Multivariable Logistic Regression Analysis of Factors Associated with Problematic Smartphone Use.

Characteristics	OR (95% CI)	*p* value
Sleep satisfaction	0.947 (0.924-0.972)	<.001
Weekend catch-up sleep (hours)		
No catch-up (≤0h) (reference)	1	
Catch-up (>0h)	1.080 (1.012-1.153)	.021
Weekday sleep duration (hours)	0.954 (0.934-0.973)	<.001
Anxiety	1.080 (1.074-1.087)	<.001
Loneliness	1.160 (1.128-1.192)	<.001
Sex		
Male (reference)	1	
Female	1.065 (1.001-1.123)	.021
Grade		
Middle school (reference)	1	
High school	0.880 (0.828-0.935)	<.001
Perceived household economic status		
High (reference)	1	
Middle	1.008 (0.960-1.060)	.741
Low	1.025 (0.950-1.107)	.521
Residential area		
Rural area (reference)	1	
Middle-sized cities	1.098 (1.008-1.197)	.032
Metropolis	1.138 (1.045-1.240)	.003
Smartphone usage time (hours)		
<4h/day (reference)	1	
≥4h/day	1.806 (1.713-1.904)	<.001
Smoking (within the last 30 days)		
No (reference)	1	
Yes	1.058 (0.935-1.196)	.370
Alcohol consumption (within the last 30 days)		
No (reference)	1	
Yes	1.074 (0.994-1.159)	.069
Physical activity (days)		
<5 days/week (reference)	1	
≥5 days/week	0.722 (0.675-0.772)	<.001
Perceived health	0.891 (0.867-0.916)	<.001

Abbreviations: OR, Odds Ratio; CI, Confidence Interval

Psychosocial factors were also strongly associated with PSU. Anxiety was positively associated with PSU (OR = 1.080, 95% CI: 1.074–1.087, *p* < .001), as was loneliness (OR = 1.160, 95% CI: 1.128–1.192, *p* < .001).

Regarding sociodemographic variables, females had higher odds of PSU than males (OR = 1.065, 95% CI: 1.001–1.123, *p* = .021). High school students had lower odds compared with middle school students (OR = 0.880, 95% CI: 0.828–0.935, *p* < .001). Residence in middle-sized cities (OR = 1.10, 95% CI: 1.01–1.20, p = .032) and metropolitan areas (OR = 1.138, 95% CI: 1.045–1.240, *p* = .003) was associated with higher odds compared with rural areas.

Smartphone use ≥ 4 hours per day was strongly associated with PSU (OR = 1.806, 95% CI: 1.713–1.904, *p* < .001). Sufficient physical activity was associated with lower odds of PSU (OR = 0.722, 95% CI: 0.675–0.772, *p* < .001). Better perceived health was also inversely associated with PSU (OR = 0.891, 95% CI: 0.867–0.916, *p* < .001). Smoking and alcohol consumption were not significantly associated with PSU after adjustment.

### Mediation analysis: role of anxiety and loneliness

Mediation analyses were conducted to examine whether anxiety and loneliness mediate the association between sleep satisfaction and PSU risk (Table 4).

**Table 4:** Mediation Effects of Anxiety and Loneliness on the Association Between Sleep Satisfaction and Problematic Smartphone Use.

Mediator	OR (95% CI)	*p* value
Anxiety		
Total effect	0.894 (0.890-0.897)	<.001
Natural direct effect	0.926 (0.923-0.928)	<.001
Natural indirect effect	0.966 (0.963-0.968)	<.001
Percentage Mediated, %	30.0	<.001
Loneliness		
Total effect	0.912 (0.909-0.915)	<.001
Natural direct effect	0.926 (0.923-0.928)	<.001
Natural indirect effect	0.986 (0.984-0.987)	<.001
Percentage Mediated, %	15.2	<.001

*Adjustments for weekend catch-up sleep, sex, grade, perceived household economic status, residential area, smoking, alcohol consumption, physical activity, and perceived health.

Abbreviations: OR, Odds Ratio; CI, Confidence Interval.

In the anxiety mediation model, the total effect of sleep satisfaction on PSU risk was significant (OR = 0.894, 95% CI: 0.890–0.897, *p* < .001). Both the natural direct effect (OR = 0.926, 95% CI: 0.923–0.928, *p* < .001) and the natural indirect effect (OR = 0.966, 95% CI: 0.963–0.968, *p* < .001) were significant. The proportion mediated was 30.0%, indicating that anxiety accounted for a substantial portion of the association between sleep satisfaction and PSU risk.

**Fig 2:**
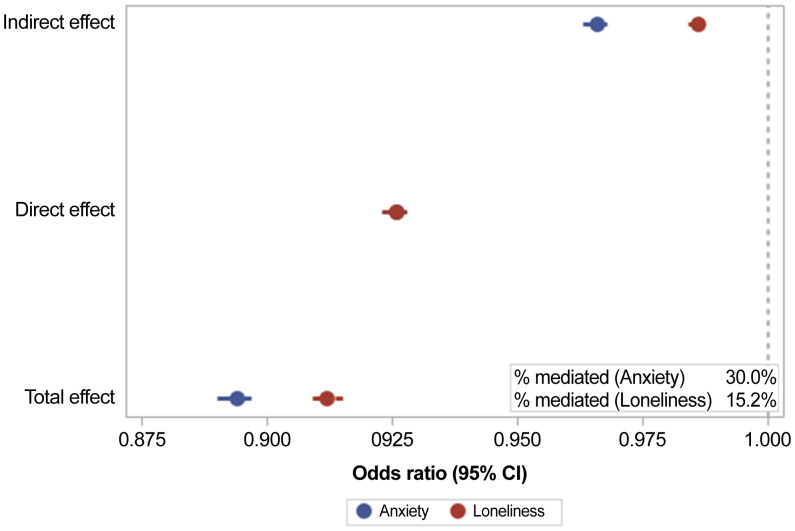
Mediation effects of anxiety and loneliness on the association between sleep satisfaction and problematic smartphone use. Points represent odds ratios (ORs) and horizontal lines indicate 95% confidence intervals (CIs). The total, natural direct, and natural indirect effects are shown for each mediator. The direct effect estimates for anxiety and loneliness overlap because they are identical.

In the loneliness mediation model, the total effect of sleep satisfaction on PSU risk was also significant (OR = 0.912, 95% CI: 0.909–0.915, *p* < .001). Both the natural direct (OR = 0.926, 95% CI: 0.923–0.928, *p* < .001) and the natural indirect effects (OR = 0.986, 95% CI: 0.984–0.987, *p* < .001) were statistically significant. The proportion mediated was 15.2%, suggesting that loneliness contributed to the association between sleep satisfaction and PSU risk. These mediation effects are visually summarized in Fig 2.

## Discussion

### Principal findings

Higher subjective sleep satisfaction in this population-based sample of Korean adolescents, was associated with lower PSU levels, and this association was partially explained by anxiety and loneliness. Notably, the indirect effect through anxiety was larger than that through loneliness. This finding suggests that, within the present parallel mediation model, emotional distress may have a relatively stronger role than social disconnection in explaining the association between lower sleep satisfaction and higher PSU among adolescents. However, this difference should be interpreted as a relative contribution within the specified statistical model, rather than as evidence that anxiety is categorically more important than loneliness in the development of PSU. Overall, these findings support the view that PSU should be understood not as an isolated behavioral problem but as part of a broader constellation of sleep-related, emotional, and social vulnerabilities in youth [22,42,43].

The inverse association between sleep satisfaction and PSU is broadly consistent with prior studies showing that problematic smartphone use is closely linked to poorer sleep and related health outcomes in adolescents. PSU in adolescents, has been associated with impaired sleep quality and health symptoms, and sleep quality has been shown to mediate the relationship between PSU and adverse health outcomes [42]. This pattern is compatible with a framework in which unsatisfactory sleep reflects not only sleep disturbance, but also reduced restoration, daytime fatigue, and diminished self-regulatory capacity. Adolescents may be more likely to rely on smartphones for distraction, mood regulation, reassurance seeking, and habitual checking, under these conditions. This study used a cross-sectional design, hence, these explanations should be interpreted as theoretically plausible mechanisms rather than empirically confirmed causal pathways. The present findings do not establish whether lower sleep satisfaction precedes PSU, whether PSU contributes to lower sleep satisfaction, or whether both are influenced by shared underlying factors. Moreover, our data did not directly assess bedtime smartphone use, nocturnal exposure, or specific motives for smartphone use. Accordingly, the observed indirect effects should be understood as model-based associations within a hypothesized framework, rather than as evidence of a deterministic causal sequence.

### Interpretation of mediation effects

The relatively larger mediating role of anxiety was one of the most distinctive findings of this study. Compared with loneliness, anxiety accounted for a greater portion of the indirect association between lower sleep satisfaction and higher PSU. This pattern suggests that emotional vulnerability may be a particularly important link in the sleep–PSU relationship among adolescents. One plausible explanation is that poor subjective sleep may reduce adolescents’ emotional regulation resources and heighten worry, rumination, irritability, and sensitivity to uncertainty. In turn, smartphones may be used as a readily available strategy for immediate relief, attentional diversion, or reassurance seeking, thereby increasing the risk of dysregulated use. Previous studies have repeatedly documented a positive association between PSU and anxiety [22], and prospective evidence indicates that increases in PSU are accompanied by an increase in anxiety symptoms and sleep-related difficulties [43]. One plausible interpretation is that poor subjective sleep reduces adolescents’ emotional regulation resources and heightens worry, rumination, irritability, and sensitivity to uncertainty. In turn, smartphones may be used as a readily available strategy for immediate relief, attentional diversion, or reassurance seeking, which may increase the risk of dysregulated use. This interpretation is particularly relevant during adolescence, when emotional reactivity, academic pressure, peer evaluation, and digital engagement often intersect. Nevertheless, this was a cross-sectional study, hence, anxiety should be interpreted as a statistically plausible intermediary rather than a definitively established causal mechanism.

The mediating role of loneliness is also theoretically meaningful in adolescence, a developmental period in which peer relationships and social belonging are particularly salient. Loneliness may increase the reward value of online interactions, validation seeking, and digitally mediated social compensation, thereby fostering greater dependence on smartphone-based communication. Thus, low sleep satisfaction may contribute to fatigue, social withdrawal, or poorer interpersonal functioning during the day, which may intensify feelings of disconnection and increase the likelihood of problematic smartphone engagement. Emerging intervention evidence is relevant here; a recent adolescent trial showed that reducing loneliness and strengthening perceived social support were accompanied by reductions in PSU [16]. This does not prove that loneliness causally mediates the present association, but rather supports the practical relevance of social connectedness as a potential intervention target.

The parallel mediation model should be interpreted with caution. Anxiety and loneliness are conceptually distinct but empirically intertwined experiences. Our model estimates their respective indirect effects within a specified statistical structure, rather than demonstrating that they operate as fully independent pathways. Therefore, this should be described as a relative contribution within the specified parallel mediation model, although anxiety showed a larger indirect effect than loneliness in the present analysis. It should not be interpreted as conclusive evidence that anxiety is categorically more important than loneliness in the development of PSU. Rather, the findings suggest that emotional distress and social disconnection may represent overlapping but distinct psychosocial processes linking sleep satisfaction to PSU. Future studies should compare alternative specifications, including models that use correlated mediators, sequential mediation, reciprocal pathways, and latent common distress factors.

A central limitation in interpreting these findings is the reverse causality. Although the model positioned sleep satisfaction as the antecedent and PSU as the outcome, the opposite direction is equally plausible. Prior longitudinal work suggests that PSU may prospectively predict worsening anxiety and sleep-related difficulties [43], whereas other longitudinal evidence indicates that poor sleep quality may precede maladaptive smartphone-related cognition [44]. The literature suggests that sleep problems and PSU may operate in a mutually reinforcing cycle rather than in a simple unidirectional chain. Therefore, the present mediation analysis should be understood as a theory-informed statistical decomposition of cross-sectional associations and not as proof of causal ordering.

Another important concern is temporal ambiguity. Mediation analysis assumes a temporal sequence from exposure to the mediator to the outcome; however, this assumption cannot be empirically verified using a single-wave design. In addition, self-reported constructs, such as sleep satisfaction, anxiety, loneliness, and PSU, may capture experiences over partially overlapping or non-equivalent recall periods, which further complicates temporal interpretation. Therefore, the direct and indirect effects observed here should be interpreted as model-based associations within a hypothesized temporal framework. Multi-wave longitudinal studies with harmonized assessment windows are necessary to determine whether low sleep satisfaction precedes increases in anxiety or loneliness, which in turn precede increases in PSU, or whether reciprocal feedback processes better explain the observed associations [43,44].

The role of smartphone use duration also warrants careful consideration. The duration of use, especially around bedtime, may act as a confounder, mediator, or component of the broader behavioral pattern captured by PSU. Prior adolescent research has indicated that sleep-related harm is particularly relevant when smartphone use intrudes into bedtime routines [42]. However, PSU is not reducible to the time spent on a device; rather, it reflects impaired control, compulsive engagement, and functional impairment. Thus, automatically adjusting for total use time may risk overadjustment if usage duration lies on the causal pathway between sleep-related vulnerability and PSU, whereas omitting it entirely may leave part of the association insufficiently characterized. Therefore, future research should distinguish between total use time, post-bedtime use, app-specific activity, and use motives to clarify whether these factors confound, mediate, or moderate the relationship between sleep satisfaction and PSU.

If a higher PSU was observed among female adolescents in the present sample, this finding may be consistent with previous work but should not be interpreted as a simple sex-based vulnerability. Differences by sex may reflect variations in communication-oriented use, social evaluative concerns, emotional coping styles, or platform preferences, rather than biological sex alone. Accordingly, sex may function not only as a covariate but also as an effect modifier. Future studies should test whether the pathways from sleep satisfaction to anxiety, loneliness, and PSU differ across sexes using stratified or multigroup models.

### Public health implications

The present findings suggest that prevention and intervention strategies that focus solely on reducing screen time may be too limited, from a public health perspective. Interventions should integrate sleep health promotion, emotional regulation support, and efforts to strengthen offline social connectedness. Existing evidence suggests that reducing bedtime smartphone use may improve sleep-related outcomes [42], and that interventions targeting loneliness and social support can also reduce PSU symptoms [16]. Moreover, prospective findings linking PSU with later anxiety and sleep problems underscore the value of addressing emotional distress alongside digital behavior [43]. This implies that sleep hygiene education, anxiety management, peer support, and family-based guidance on device use may be more effective when implemented as complementary rather than as isolated strategies, for schools and community settings. However, these implications remain hypothesized in the context of the present cross-sectional study and should be tested in longitudinal and interventional research.

In summary, this study indicated that subjective sleep satisfaction, anxiety, loneliness, and PSU are closely interconnected among Korean adolescents. Anxiety and loneliness accounted for part of the association between lower sleep satisfaction and higher PSU, with anxiety showing a relatively larger indirect effect than loneliness within the parallel mediation model. Nonetheless, these findings should be interpreted cautiously because cross-sectional mediation cannot establish a temporal or causal direction, and reciprocal relationships among sleep, emotional distress, social disconnection, and PSU remain likely [43,44]. Future research should use repeated-measures longitudinal designs and more refined behavioral indicators of smartphone use to clarify directionality, distinguish overlapping psychosocial mechanisms, and identify the most effective targets for adolescent PSU prevention.

### Limitations and future directions

This study has several limitations that should be considered when interpreting the findings. First, owing to the cross-sectional design, causal relationships could not be established, and the temporal ordering between sleep satisfaction, anxiety, loneliness, and PSU could not be confirmed. Therefore, reverse causality remains possible; for example, PSU may contribute to lower sleep satisfaction, anxiety symptoms, or loneliness, rather than only the reverse. Future longitudinal studies are needed to clarify the directionality and possible reciprocal relationships among sleep satisfaction, emotional distress, social disconnection, and PSU.

Second, the KYRBS relies on self-reported responses; therefore, recall bias, reporting bias, and social desirability bias cannot be completely excluded. In particular, adolescents may have underreported or overreported sleep-related behaviors, emotional experiences, or smartphone use.

Third, sleep satisfaction and loneliness were assessed using single-item measures. Although single-item measures can be useful in large-scale population-based surveys and have been used as brief global indicators, they may not fully capture the multidimensional nature of sleep satisfaction and loneliness. Therefore, measurement error and limited content validity should be considered when interpreting these findings. Future studies should use validated multi-item instruments to assess sleep quality, sleep satisfaction, loneliness, and related psychosocial constructs more comprehensively.

Fourth, although we adjusted for available sociodemographic and health-related covariates, residual confounding may remain. Some potentially important contextual and individual factors, including detailed family structure, parental monitoring, school-level characteristics, sexual identity, cultural background, deprivation or inequality indicators, and clinically diagnosed comorbid mental health conditions, were not available in the secondary dataset and therefore could not be fully adjusted for in this study. Future research should incorporate more comprehensive individual, family, school, and sociocultural variables to better account for potential confounding.

Fifth, since this study was conducted among Korean adolescents, whose sleep patterns, academic demands, and digital environments may differ from those in other cultural contexts, caution is needed when generalizing these findings to other populations.

## Conclusions

Higher sleep satisfaction in Korean adolescents, was associated with a lower PSU risk, and this association was significantly and partially mediated by anxiety and loneliness. The relatively greater contribution of anxiety suggests that PSU may reflect not only excessive smartphone use but also the interplay between sleep-related recovery and emotional vulnerability. These findings highlight the importance of considering sleep health and psychosocial factors to understand PSU risks. Prevention strategies may benefit from integrated approaches, including promoting healthy sleep behaviors, supporting emotional regulation, and strengthening social connectedness, from a public health perspective. Future longitudinal studies are needed to clarify the temporal relationships and potential bidirectional interactions between sleep satisfaction, anxiety, loneliness, and PSU.

## Supporting information

S1 Table Mediation Effects of Anxiety and Loneliness on the Association Between Sleep Satisfaction and Problematic Smartphone Use. Description of data: Sensitivity Analyses of the Mediation Effects of Anxiety and Loneliness on the Association Between Sleep Satisfaction and Problematic Smartphone Use.
(DOCX)

S1 Checklist STROBE-checklist-v4-cross-sectional. (DOC)
