# Supplementary material for: Sleep satisfaction and problematic smartphone use among adolescents in the Republic of Korea: The mediating roles of anxiety and loneliness
Source: PLoS One. 2026 Jul 24;21(7):e0354423. doi: 10.1371/journal.pone.0354423 (PMC13399282; doi:10.1371/journal.pone.0354423)
Supplement: S1 Table — Description of data: Sensitivity Analyses of the Mediation Effects of Anxiety and Loneliness on the Association Between Sleep Satisfaction and Problematic Smartphone Use. (DOCX) [file pone.0354423.s001.docx]

**Supplementary Table S1.** Mediation Effects of Anxiety and Loneliness on the Association Between Sleep Satisfaction and Problematic Smartphone Use

| **Mediator** | **OR (95% CI)** | ***p* value** |
| --- | --- | --- |
| Anxiety |  |  |
| Total effect | 0.918 (0.914-0.922) | <.001 |
| Natural direct effect | 0.948 (0.945-0.951) | <.001 |
| Natural indirect effect | 0.969 (0.966-0.971) | <.001 |
| Percentage Mediated, % | 35.9 | <.001 |
| Loneliness |  |  |
| Total effect | 0.935 (0.932-0.939) | <.001 |
| Natural direct effect | 0.948 (0.945-0.951) | <.001 |
| Natural indirect effect | 0.987 (0.986-0.988) | <.001 |
| Percentage Mediated, % | 18.8 | <.001 |

*Adjustments for weekend catch-up sleep, weekday sleep duration, sex, grade, Perceived household economic status, residential area, smartphone usage time, smoking, alcohol consumption, physical activity, and perceived health.

Abbreviations: OR, Odds Ratio; CI, Confidence Interval.
