# Supplementary material for: Sleep satisfaction and problematic smartphone use among adolescents in the Republic of Korea: The mediating roles of anxiety and loneliness
Source: PLoS One. 2026 Jul 24;21(7):e0354423. doi: 10.1371/journal.pone.0354423 (PMC13399282; doi:10.1371/journal.pone.0354423)
Supplement: Attachment — Submitted filename: Responding_to_peer_reviewer_comments__1_2_0613.docx [file pone.0354423.s003.docx]

Dear Dr. Fucai Lin and reviewers,

Thank you for giving us the opportunity to submit a revised draft of our manuscript, now titled “Sleep satisfaction and problematic smartphone use among adolescents in the Republic of Korea: The mediating roles of anxiety and loneliness” to PLOS ONE. We appreciate the time and effort that the reviewers and you have dedicated to providing valuable feedback on our manuscript. Their insightful and constructive comments have helped us improve the clarity, methodological rigor, and theoretical grounding of the paper.

We have carefully revised the manuscript to address the points raised by the reviewers. The major revisions include clarification of the study context in the Title and the Methods section, strengthening of the theoretical framework, clarification of the cross-sectional nature of the mediation analysis, additional discussion of residual confounding and reverse causality, expansion of the Limitations section, clarification of the data availability statement, correction and verification of references, and revision of the manuscript structure. We have also highlighted all changes in the revised manuscript.

Here is a point-by-point response to the reviewers’ comments and concerns.

**Comments from Reviewer 1**

- Comment 1: Thanks so much for the submission. I enjoyed reading through this well prepared manuscript. Please can I clarify - title could be made clearer as to suggest that this is a case study of a specific context i.e. a case study of adolescents in the Republic of Korea (this enables the reader conceptualise possible systemic factors contributing to results).

Response: Thank you for your positive evaluation of our manuscript and for this helpful suggestion. We agree that the title should clearly indicate the specific national context of the study. Accordingly, we have revised the title to clarify that the study was conducted among adolescents in the Republic of Korea.

The revised title is “Sleep satisfaction and problematic smartphone use among adolescents in the Republic of Korea: The mediating roles of anxiety and loneliness.” This change has been made on the title page and throughout the revised manuscript where appropriate.

- Comment 2: Can I check that the article outlines described consent meets the legal age of consent for the context, whether there were any incentives for response or even deterrents, whether there could be the possibility of recall /observer bias in responses and if there was any consideration of standardisation for confounders in responses such as cultural, deprivation/inequality, home situation i.e. single parent, sexuality, comorbid mental illness etc.; sadly as I am ignorant of this context are all the educational institutions rated of the same standard maybe?

Response: Thank you for raising these important ethical and methodological issues. We agree that the manuscript needed clearer information regarding the consent procedure, voluntary participation, incentives or deterrents, potential response bias, and consideration of confounding factors.

In response to this comment, we have revised the Materials and methods – Participants section to clarify that this study was based on a secondary analysis of data from the 19th Korea Youth Risk Behavior Survey (KYRBS), a government-approved national statistical survey jointly administered by the Korea Disease Control and Prevention Agency and the Ministry of Education. We have clarified that the original survey was an anonymous, self-administered, web-based survey conducted among middle and high school students nationwide. Before participation, students were informed of the survey purpose, procedures, voluntary nature of participation, anonymity, and confidentiality. Students participated only if they agreed to complete the survey, and they could refuse or discontinue participation without penalty. Because the present study used only de-identified, publicly available secondary data, the researchers had no access to personally identifiable information.

We have also clarified that, based on the publicly available survey documentation, no individual-level incentives or deterrents related to participation were reported. In addition, we have added a statement in the Discussion section acknowledging that, because the KYRBS relies on self-reported responses, recall bias, reporting bias, and social desirability bias cannot be completely excluded.

Regarding confounding factors, we have revised the manuscript to specify the available sociodemographic and health-related variables included as covariates in the adjusted analyses, such as sex, school grade, academic achievement, perceived economic status, living arrangement, and perceived health status. We have also acknowledged that some potentially important contextual and individual factors, including detailed family structure, parental monitoring, school-level characteristics, sexual identity, cultural background, deprivation or inequality indicators, and clinically diagnosed comorbid mental health conditions, were not available in the secondary dataset and therefore could not be fully adjusted for in this study.

These revisions have been incorporated into the Materials and methods – Participants section and the Discussion – Limitations section of the revised manuscript.

- Comment 3: The approach and the results are plausible and seem to be in keeping with the evidence shared however what was the unusual finding that stood out for you and why?

Response: Thank you for this insightful comment. We agree that the Discussion section could more clearly identify and interpret the finding that stood out most in our results.

In the revised manuscript, we have clarified that the most notable finding was that the indirect pathway through anxiety appeared larger than the pathway through loneliness. This finding was noteworthy because both anxiety and loneliness were significant mediators, but anxiety showed a relatively greater contribution within the parallel mediation model. We interpreted this as suggesting that emotional distress may be a particularly salient correlate of the association between lower sleep satisfaction and higher problematic smartphone use among adolescents.

We have further revised the Discussion to explain why this finding may be meaningful in adolescence. Poor subjective sleep satisfaction may reduce emotional regulation capacity and increase worry, rumination, irritability, and sensitivity to uncertainty. In this context, smartphones may be used as an easily accessible means of distraction, reassurance seeking, or short-term mood regulation, which may increase vulnerability to dysregulated use. At the same time, we have clarified that this result should not be interpreted as evidence that anxiety is categorically more important than loneliness, because the study used cross-sectional data and the mediators were examined within a specified statistical model.

These revisions have been incorporated into the Discussion section.

- Comment 4: Would you be able to opine with regards to current results if anxiety had a lower mediation effect than loneliness or not?

Response: Thank you for this valuable comment. We agree that the relative magnitude of the mediation effects could be interpreted more clearly. In the revised Discussion, we have clarified that the indirect effect through anxiety was larger than that through loneliness in the present parallel mediation model. This finding suggests that emotional distress may have a relatively stronger role than social disconnect on in explaining the association between lower sleep satisfaction and higher PSU among adolescents.

We have further suggested that poor subjective sleep may reduce adolescents’ emotional regulation resources and heighten worry, rumination, irritability, and sensitivity to uncertainty. In this context, smartphones may be used as a readily available strategy for immediate relief, attentional diversion, or reassurance seeking, which may increase the risk of dysregulated use.

At the same time, we have revised the Discussion to emphasize that this difference should be interpreted cautiously. The larger indirect effect through anxiety reflects a relative contribution within the specified statistical model and should not be interpreted as conclusive evidence that anxiety is categorically more important than loneliness in the development of PSU. We have also noted that anxiety and loneliness may represent overlapping but distinct psychosocial processes linking sleep satisfaction to PSU.

- Comment 5: I wonder if there are any other ways that the data can be approached and which you considered and discarded as well?

Response: Thank you for this thoughtful question. We are happy to justify our analytical approach and acknowledge the possible alternative approaches. During preliminary analyses, PSU was also examined as a continuous outcome using the total score of the Problematic Smartphone Use Screening Tool (PSS) in exploratory analyses. The overall pattern of findings was consistent with that observed in the primary analyses using PSU as a dichotomous outcome. Lower sleep satisfaction, higher anxiety, and higher loneliness remained significantly associated with greater PSU severity. However, because the primary objective of this study was to identify adolescents at elevated risk of problematic smartphone use and because the PSS was specifically developed as a screening instrument with established cut-off values, we retained the dichotomous PSU outcome for the primary analyses. We have clarified this rationale in the revised manuscript.

- Comment 6: Would you like to consider itemising subheadings such as limitations or applications and recommendations as separate aspects rather than embed within discussion and conclusion maybe?

Response: Thank you for this helpful suggestion. We agree that the Discussion section would be clearer if the limitations, implications, and recommendations were presented under separate subheadings.

Accordingly, we have restructured the Discussion section using the following subheadings: Principal findings, Interpretation of mediation effects, Public health implications, Limitations and future directions. This revision improves the readability and organization of the manuscript.

- Comment 7: I struggled to access references 17 and 30 which you outlined as published in 2025 but which my generic search brings up as 2023 - is this an error or are these papers blocked in my context maybe?

Response: Thank you for carefully checking these references. We have reviewed References 17 and 30 and confirmed that both references are correctly cited as 2025 publications in the revised manuscript. The year 2023 refers to the online first publication date, whereas the final issue publication information is 2025. Therefore, we retained 2025 as the publication year in the reference list, following the final volume, issue, and page information.

To avoid ambiguity, we rechecked the bibliographic details, including authors, title, journal name, volume, issue, page numbers, and DOI, and confirmed that the reference information is accurate.

**Comments from Reviewer 2**

- Comment 1: Cross-sectional mediation cannot establish causal pathways.

Response: Thank you for this important comment. We fully agree that mediation analysis based on cross-sectional data, cannot establish causal pathways or confirm temporal ordering among sleep satisfaction, anxiety, loneliness, and PSU.

In response to this comment, we have revised the Discussion to clarify that the proposed mechanisms should be interpreted as theoretically plausible explanations, rather than as empirically confirmed causal pathways. We have also added a statement that the present findings do not establish whether lower sleep satisfaction precedes PSU, whether PSU contributes to lower sleep satisfaction, or whether both are influenced by shared underlying factors. In addition, we have clarified that the observed mediation effects should be understood as model-based indirect associations within a hypothesized framework, rather than as evidence of a deterministic causal sequence.

We have also revised causal wording throughout the manuscript to ensure that the findings are presented as associations rather than causal effects.

- Comment 2: Reliance on single-item measures for sleep satisfaction and loneliness.

Response: Thank you for pointing out this important measurement issue. We agree that the use of single-item measures for sleep satisfaction and loneliness is a limitation of this study.

In response to this comment, we have revised the Measures section to provide a clearer description of how sleep satisfaction and loneliness were assessed. Specifically, we have explained that sleep satisfaction was measured using a single self-reported item asking whether sleep during the past 7 days was sufficient to recover from fatigue. We have also clarified that loneliness was measured using a single item asking how often participants felt lonely during the past 12 months. Although single-item measures can be useful in large-scale population-based surveys and have been used as brief global indicators, we recognize that they may not fully capture the multidimensional nature of sleep satisfaction and loneliness.

Accordingly, we have added this issue to the Limitations section. We have stated that measurement error and limited content validity should be considered when interpreting the findings, and that future studies should use validated multi-item instruments to assess sleep quality, sleep satisfaction, loneliness, and related psychosocial constructs more comprehensively.

- Comment 3: Insufficient justification for dichotomizing PSU.

Response: Thank you for this important comment. We agree that dichotomizing a behavioral scale can lead to loss of information and should be justified carefully. In the revised manuscript, we have strengthened the rationale for operationalizing problematic smartphone use (PSU) as a binary risk indicator

First, our decision was based on the purpose and design of the PSS itself, which was developed as a screening instrument for identifying adolescents at risk of smartphone overdependence in the Korean public health context, rather than as a purely dimensional research scale. The cutoffs distinguishing general users, potential-risk users, and high-risk users were established in prior national surveillance practice, and have been used in the Korea Youth Risk Behavior Survey framework. Thus, combining the potential-risk and high-risk groups into a single “PSU risk” category is consistent with the tool’s intended screening use and public health application.

Second, this categorization aligns with the broader literature showing that PSU is often conceptualized not simply as high frequency of use, but as a clinically and functionally meaningful pattern involving impaired control, salience, and negative consequences (Elhai et al., 2019) . In that sense, the screening threshold is useful for identifying adolescents whose smartphone use may already be associated with psychosocial vulnerability and functional impairment.

Third, this operationalization also improves the interpretability and policy relevance of our findings. Our study aims to identify adolescents at elevated risk who may benefit from prevention or intervention, hence, a binary classification provides results that are more directly translatable into school health screening and public health strategies. This is particularly relevant in Korean adolescent populations, where nationally representative studies have also used threshold-based approaches to identify risk patterns in smartphone-related behaviors and mental health outcomes (Woo et al., 2021) .

- Comment 4: Need for stronger theoretical framework and must incorporate this fully.

Response: Thank you for this important suggestion. We agree that the theoretical framework of the study needed to be strengthened and more fully incorporated into the manuscript.

In response to this comment, we have substantially revised the Introduction to provide a clearer theoretical rationale for examining sleep satisfaction, anxiety, loneliness, and PSU within a psychosocial vulnerability framework. Specifically, we conceptualized PSU not merely as excessive smartphone use, but as a maladaptive pattern of use that may emerge when adolescents rely on smartphones to cope with emotional discomfort, unmet interpersonal needs, or weakened self-regulation.

To support this rationale, we have incorporated the Compensatory Internet Use Theory and the I-PACE model, both of which explain how emotional distress, cognitive-affective responses, and impaired self-regulation may contribute to problematic technology use. We have also added relevant literature on the role of anxiety in PSU, the distinction between PSU and smartphone use duration, the distinction between PSU and post-bedtime smartphone use, and the social-affective pathway linking loneliness to problematic internet or smartphone use.

We have further clarified why sleep satisfaction was treated as a theoretically meaningful antecedent condition, rather than only as an outcome of smartphone behavior. We have explained that low sleep satisfaction may reflect reduced restoration, fatigue, irritability, and weakened emotion regulation resources, which may increase adolescents’ vulnerability to anxiety, loneliness, and maladaptive smartphone engagement.

Finally, we have revised the Introduction to present loneliness and anxiety as complementary parallel mediators. Loneliness was framed as a social-affective pathway, whereas anxiety was framed as an emotional regulation pathway. We have also added a clear statement that reverse or reciprocal pathways remain plausible, and that the proposed model should be interpreted as a theory-driven mediation model rather than as evidence of causal ordering.

- Comment 5: Figure mentioned missing.

Response: Thank you for identifying this issue. we have added Fig 1, which presents the participant selection process, and Fig 2, which presents the hypothesized parallel mediation model linking sleep satisfaction, anxiety, loneliness, and problematic smartphone use. Both figures have been uploaded as separate figure files according to the journal’s submission requirements.

- Comment 6: Hypotheses and null hypotheses incomplete.

Response: Thank you for this helpful comment. We agree that the hypotheses and null hypotheses were not sufficiently specified in the previous version of the manuscript.

In response to this comment, we have revised the final part of the Introduction section to clearly present both the research hypotheses and the corresponding null hypotheses. Specifically, we have added hypotheses regarding the association between sleep satisfaction and PSU risk, the associations between sleep satisfaction and the two proposed mediators, and the indirect effects through loneliness and anxiety symptoms.

- Comment 7: Methodology section silent on setting, tool, description, techniques of sampling.

Response: Thank you for this important comment. We agree that the Methods section required more detailed information about the study setting, data source, survey tool, sample description, and sampling method.

We have substantially revised the Methods section to include the following information: study design, national setting, data source, original survey procedure, sampling strategy, inclusion and exclusion criteria, final analytic sample, survey instruments, variable definitions, and statistical techniques. We have clarified that this study was a secondary analysis of nationally collected cross-sectional survey data from adolescents in the Republic of Korea.

We have also added information about the sampling design, including stratification and cluster sampling where applicable, and clarified whether survey weights were applied in the analysis.

- Comment 8: Limited discussion of residual confounding and reverse causality.

Response: Thank you for this important observation. We agree that the Discussion section should have more explicitly addressed residual confounding and reverse causality.

In response to this comment, we have revised the Limitations and future directions section to clarify that, owing to the cross-sectional design, causal relationships could not be established and the temporal ordering between sleep satisfaction, anxiety, loneliness, and PSU could not be confirmed. We have also added that reverse causality remains possible; for example, PSU may contribute to lower sleep satisfaction, anxiety symptoms, or loneliness, rather than only the reverse.

In addition, we have expanded the discussion of residual confounding. Although our analyses adjusted for available sociodemographic and health-related covariates, some potentially important contextual and individual factors, including detailed family structure, parental monitoring, school-level characteristics, sexual identity, cultural background, deprivation or inequality indicators, and clinically diagnosed comorbid mental health conditions, were not available in the secondary dataset and therefore could not be fully adjusted for in this study.

We have also revised causal wording throughout the manuscript to ensure that the findings are presented as associations and model-based indirect effects rather than causal pathways.

- Comment 9: Cohen’s D missing.

Response: Thank you for this comment. Cohen's D is typically used to quantify standardized mean differences between two groups. The primary analyses in this study consisted of survey-weighted logistic regression and causal mediation models. Therefore, we reported effect estimates that are more appropriate for these analytical approaches, including odds ratios, confidence intervals, standardized coefficients, and indirect effect estimates.

- Comment 10: Study likely falls in SMRA as is based on secondary data analysis.

Response: Thank you for this helpful comment. We agree that the manuscript should clearly report its nature as a secondary analysis of existing survey data and follow appropriate reporting standards for observational studies.

In response to this comment, we have revised the Materials and methods section to explicitly state that this study was a secondary analysis of data from the 19th Korea Youth Risk Behavior Survey, a nationally representative cross-sectional survey of Korean adolescents. We have also strengthened the reporting of the study design, setting, participants, sampling procedures, variables, data source, potential sources of bias, and statistical methods.

In addition, we have reviewed the manuscript according to the STROBE reporting guideline for cross-sectional studies and submitted the completed STROBE checklist as a supplementary file. This checklist indicates where each relevant reporting item is addressed in the revised manuscript.
