# Supplementary material for: Sleep satisfaction and problematic smartphone use among adolescents in the Republic of Korea: The mediating roles of anxiety and loneliness
Source: PLoS One. 2026 Jul 24;21(7):e0354423. doi: 10.1371/journal.pone.0354423 (PMC13399282; doi:10.1371/journal.pone.0354423)
Supplement: Peer review history

# Peer review: Decision Letter 0

**DOI:** 10.1371/journal.pone.0354423.r001

**Authors:** Fucai Lin

4 Jun 2026

PONE-D-26-24249

The mediating roles of anxiety and loneliness in the relationship between sleep satisfaction and problematic smartphone use among adolescents

PLOS One

Dear Dr. Cho,

Thank you for submitting your manuscript to PLOS ONE. After careful consideration, we feel that it has merit but does not fully meet PLOS ONE’s publication criteria as it currently stands. Therefore, we invite you to submit a revised version of the manuscript that addresses the points raised during the review process.

Please submit your revised manuscript by Jul 04 2026 11:59PM. If you will need more time than this to complete your revisions, please reply to this message or contact the journal office at plosone@plos.org. When you're ready to submit your revision, log on to https://www.editorialmanager.com/pone/ and select the 'Submissions Needing Revision' folder to locate your manuscript file.

Please include the following items when submitting your revised manuscript:

- A letter that responds to each point raised by the academic editor and reviewer(s). You should upload this letter as a separate file labeled 'Response to Reviewers'.
- A marked-up copy of your manuscript that highlights changes made to the original version. You should upload this as a separate file labeled 'Revised Manuscript with Track Changes'.
- An unmarked version of your revised paper without tracked changes. You should upload this as a separate file labeled 'Manuscript'.

If you would like to make changes to your financial disclosure, please include your updated statement in your cover letter. Guidelines for resubmitting your figure files are available below the reviewer comments at the end of this letter.

If applicable, we recommend that you deposit your laboratory protocols in protocols.io to enhance the reproducibility of your results. Protocols.io assigns your protocol its own identifier (DOI) so that it can be cited independently in the future. For instructions see: https://journals.plos.org/plosone/s/submission-guidelines#loc-laboratory-protocols. Additionally, PLOS ONE offers an option for publishing peer-reviewed Lab Protocol articles, which describe protocols hosted on protocols.io. Read more information on sharing protocols at https://plos.org/protocols?utm_medium=editorial-email&utm_source=authorletters&utm_campaign=protocols.

As the corresponding author, your ORCID iD is verified in the submission system and will appear in the published article. PLOS supports the use of ORCID, and we encourage all coauthors to register for an ORCID iD and use it as well. Please encourage your coauthors to verify their ORCID iD within the submission system before final acceptance, as unverified ORCID iDs will not appear in the published article. *Only*  the individual author can complete the verification step; PLOS staff *cannot*  verify ORCID iDs on behalf of authors.

We look forward to receiving your revised manuscript.

Kind regards,

Fucai Lin, Ph.D.

Academic Editor

PLOS One

Journal Requirements:

When submitting your revision, we need you to address these additional requirements.

1. Please ensure that your manuscript meets PLOS ONE's style requirements, including those for file naming. The PLOS ONE style templates can be found at https://journals.plos.org/plosone/s/file?id=wjVg/PLOSOne_formatting_sample_main_body.pdf and https://journals.plos.org/plosone/s/file?id=ba62/PLOSOne_formatting_sample_title_authors_affiliations.pdf

2. If the reviewer comments include a recommendation to cite specific previously published works, please review and evaluate these publications to determine whether they are relevant and should be cited. There is no requirement to cite these works unless the editor has indicated otherwise.

3. Please review your reference list to ensure that it is complete and correct. If you have cited papers that have been retracted, please include the rationale for doing so in the manuscript text, or remove these references and replace them with relevant current references. Any changes to the reference list should be mentioned in the rebuttal letter that accompanies your revised manuscript. If you need to cite a retracted article, indicate the article’s retracted status in the References list and also include a citation and full reference for the retraction notice.

[Note: HTML markup is below. Please do not edit.]

Reviewers' comments:

Reviewer's Responses to Questions

**Comments to the Author**

1. Is the manuscript technically sound, and do the data support the conclusions?

The manuscript must describe a technically sound piece of scientific research with data that supports the conclusions. Experiments must have been conducted rigorously, with appropriate controls, replication, and sample sizes. The conclusions must be drawn appropriately based on the data presented.

Reviewer #1: Yes

Reviewer #2: Yes

**********

2. Has the statistical analysis been performed appropriately and rigorously?

Reviewer #1: Yes

Reviewer #2: Yes

**********

3. Have the authors made all data underlying the findings in their manuscript fully available?

The PLOS Data policy requires authors to make all data underlying the findings described in their manuscript fully available without restriction, with rare exception (please refer to the Data Availability Statement in the manuscript PDF file). The data should be provided as part of the manuscript or its supporting information, or deposited to a public repository. For example, in addition to summary statistics, the data points behind means, medians and variance measures should be available. If there are restrictions on publicly sharing data—e.g. participant privacy or use of data from a third party—those must be specified.

Reviewer #1: Yes

Reviewer #2: No

**********

4. Is the manuscript presented in an intelligible fashion and written in standard English?

PLOS ONE does not copyedit accepted manuscripts, so the language in submitted articles must be clear, correct, and unambiguous. Any typographical or grammatical errors should be corrected at revision, so please note any specific errors here.

Reviewer #1: Yes

Reviewer #2: Yes

**********

5. Review Comments to the Author

Please use the space provided to explain your answers to the questions above. You may also include additional comments for the author, including concerns about dual publication, research ethics, or publication ethics. (Please upload your review as an attachment if it exceeds 20,000 characters)

Reviewer #1: Thanks so much for the submission . I enjoyed reading through this well prepared manuscript. Please can I clarify - title could be made clearer as to suggest that this is a case study of a specific context i.e. a case study of adolescents in the Republic of Korea ( this enables the reader conceptualise possible systemic factors contributing to results). Can I check that the article outlines described consent meets the legal age of consent for the context , whether there were any incentives for response or even deterrents, whether there could be the possibility of recall /observer bias in responses and if there was any consideration of standardisation for confounders in responses (such as cultural, deprivation/inequality, home situation i.e. single parent, sexuality, comorbid mental illness etc; sadly as I am ignorant of this context are all the educational institutions rated of the same standard maybe?). The approach and the results are plausible and seem to be in keeping with the evidence shared however what was the unusual finding that stood out for you and why? Would you be able to opine with regards to current results if anxiety had a lower mediation effect than loneliness or not? I wonder if there are any other ways that the data can be approached and which you considered and discarded as well?

Would you like to consider itemising subheadings such as limitations or applications and recommendations as separate aspects rathe than embed within discussion and conclusion maybe?

I struggled to access references 17 and 30 which you outlined as published in 2025 but which my generic search brings up as 2023 - is this an error or are these papers blocked in my context maybe?

I must say I really enjoyed reading the article and appreciate the overt and subtle suggestions sprinkled in its writing- cant wait to se it in print and to hear further about the follow-on research. Thanks again for sharing.

Reviewer #2: Cross-sectional mediation cannot establish causal pathways.

Reliance on single-item measures for sleep satisfaction and loneliness.

Insufficient justification for dichotomizing PSU.

Need for stronger theoretical framework and must incorporate this fully

Figure mentioned missing

Hypotheses and null hypotheses incomplete

Methodology section silent on setting, tool, description, techniques of sampling

Limited discussion of residual confounding and reverse causality.

Cohens D missing

Study likely falls in SMRA as is based on secondary data analysis

**********

6. PLOS authors have the option to publish the peer review history of their article (what does this mean?). If published, this will include your full peer review and any attached files.

If you choose “no”, your identity will remain anonymous but your review may still be made public.

**Do you want your identity to be public for this peer review?**  For information about this choice, including consent withdrawal, please see our Privacy Policy.

Reviewer #1: **Yes:**  Olubunmi Arogunmati

Reviewer #2: **Yes:**  Noorul Amin

**********

[NOTE: If reviewer comments were submitted as an attachment file, they will be attached to this email and accessible via the submission site. Please log into your account, locate the manuscript record, and check for the action link "View Attachments". If this link does not appear, there are no attachment files.]

To ensure your figures meet our technical requirements, please review our figure guidelines: https://journals.plos.org/plosone/s/figures

You may also use PLOS’s free figure tool, NAAS, to help you prepare publication quality figures: https://journals.plos.org/plosone/s/figures#loc-tools-for-figure-preparation.

NAAS will assess whether your figures meet our technical requirements by comparing each figure against our figure specifications.

# Author comment: Author response to Decision Letter 1

**DOI:** 10.1371/journal.pone.0354423.r002

26 Jun 2026

Dear Editor:

We wish to re-submit the manuscript titled “Sleep satisfaction and problematic smartphone use among adolescents in the Republic of Korea: The mediating roles of anxiety and loneliness.” The manuscript ID is PONE-D-26-24249.

We thank you and the reviewers for your thoughtful suggestions and insights. The manuscript has benefited from these insightful suggestions. I look forward to working with you and the reviewers to move this manuscript closer to publication in PLOS One.

The manuscript has been rechecked and the necessary changes have been made in accordance with the reviewers’ suggestions. The responses to all comments have been prepared and attached herewith.

Thank you for your consideration. I look forward to hearing from you.

Attachment Submitted filename: Responding_to_peer_reviewer_comments__1_2_0613.docx

# Peer review: Decision Letter 1

**DOI:** 10.1371/journal.pone.0354423.r003

**Authors:** Fucai Lin

8 Jul 2026

Sleep satisfaction and problematic smartphone use among adolescents in the Republic of Korea: The mediating roles of anxiety and loneliness

PONE-D-26-24249R1

Dear Dr. Cho,

We’re pleased to inform you that your manuscript has been judged scientifically suitable for publication and will be formally accepted for publication once it meets all outstanding technical requirements.

Within one week, you’ll receive an e-mail detailing the required amendments. When these have been addressed, you’ll receive a formal acceptance letter and your manuscript will be scheduled for publication.

An invoice will be generated when your article is formally accepted. Please note, if your institution has a publishing partnership with PLOS and your article meets the relevant criteria, all or part of your publication costs will be covered. Please make sure your user information is up-to-date by logging into Editorial Manager at Editorial Manager® and clicking the ‘Update My Information' link at the top of the page. For questions related to billing, please contact billing support.

If your institution or institutions have a press office, please notify them about your upcoming paper to help maximize its impact. If they’ll be preparing press materials, please inform our press team as soon as possible -- no later than 48 hours after receiving the formal acceptance. Your manuscript will remain under strict press embargo until 2 pm Eastern Time on the date of publication. For more information, please contact onepress@plos.org.

Kind regards,

Fucai Lin, Ph.D.

Academic Editor

PLOS One

Additional Editor Comments (optional):

-->Review Comments to the Author

Reviewer #1: Thanks for taking on board previous comments and providing explanations where not applied. I feel this is a well presented and logical argument that sets the tone and stage for further exploration and discussion about the subject matter especially in current climate of restriction of social media in children and young people as a protective public health and preventative mental health measure. It would be interesting to see how the wider public, policy makers, practitioners and researchers/academicians perceive what it suggests and if it acts as a point of galvanising social action to protect the next generation. Thanks again for your effort.

Reviewer #2: (No Response)

**********

# Editor report: Acceptance letter

**DOI:** 10.1371/journal.pone.0354423.r004

**Authors:** Fucai Lin

PONE-D-26-24249R1

PLOS One

Dear Dr. Cho,

I'm pleased to inform you that your manuscript has been deemed suitable for publication in PLOS One. Congratulations! Your manuscript is now being handed over to our production team.

At this stage, our production department will prepare your paper for publication. This includes ensuring the following:

* All references, tables, and figures are properly cited

* All relevant supporting information is included in the manuscript submission,

* There are no issues that prevent the paper from being properly typeset

You will receive further instructions from the production team, including instructions on how to review your proof when it is ready. Please keep in mind that we are working through a large volume of accepted articles, so please give us a few days to review your paper and let you know the next and final steps.

Lastly, if your institution or institutions have a press office, please let them know about your upcoming paper now to help maximize its impact. If they'll be preparing press materials, please inform our press team within the next 48 hours. Your manuscript will remain under strict press embargo until 2 pm Eastern Time on the date of publication. For more information, please contact onepress@plos.org.

You will receive an invoice from PLOS for your publication fee after your manuscript has reached the completed accept phase. If you receive an email requesting payment before acceptance or for any other service, this may be a phishing scheme. Learn how to identify phishing emails and protect your accounts at https://explore.plos.org/phishing.

If we can help with anything else, please email us at customercare@plos.org.

Thank you for submitting your work to PLOS ONE and supporting open access.

Kind regards,

PLOS ONE Editorial Office Staff

on behalf of

Professor Fucai Lin

Academic Editor

PLOS One
